# Genetic linkage map of a wild genome: genomic structure, recombination and sexual dimorphism in bighorn sheep

**DOI:** 10.1186/1471-2164-11-524

**Published:** 2010-09-28

**Authors:** Jocelyn Poissant, John T Hogg, Corey S Davis, Joshua M Miller, Jillian F Maddox, David W Coltman

**Affiliations:** 1 Department of Biological Sciences, University of Alberta, Edmonton, Alberta, T6G 2E9, Canada; 2 Montana Conservation Science Institute, 5200 Upper Miller Creek Road, Missoula, MT 59803, USA; 3 Department of Veterinary Science, University of Melbourne, Victoria 3010, Australia

## Abstract

**Background:**

The construction of genetic linkage maps in free-living populations is a promising tool for the study of evolution. However, such maps are rare because it is difficult to develop both wild pedigrees and corresponding sets of molecular markers that are sufficiently large. We took advantage of two long-term field studies of pedigreed individuals and genomic resources originally developed for domestic sheep (*Ovis aries*) to construct a linkage map for bighorn sheep, *Ovis canadensis*. We then assessed variability in genomic structure and recombination rates between bighorn sheep populations and sheep species.

**Results:**

Bighorn sheep population-specific maps differed slightly in contiguity but were otherwise very similar in terms of genomic structure and recombination rates. The joint analysis of the two pedigrees resulted in a highly contiguous map composed of 247 microsatellite markers distributed along all 26 autosomes and the X chromosome. The map is estimated to cover about 84% of the bighorn sheep genome and contains 240 unique positions spanning a sex-averaged distance of 3051 cM with an average inter-marker distance of 14.3 cM. Marker synteny, order, sex-averaged interval lengths and sex-averaged total map lengths were all very similar between sheep species. However, in contrast to domestic sheep, but consistent with the usual pattern for a placental mammal, recombination rates in bighorn sheep were significantly greater in females than in males (~12% difference), resulting in an autosomal female map of 3166 cM and an autosomal male map of 2831 cM. Despite differing genome-wide patterns of heterochiasmy between the sheep species, sexual dimorphism in recombination rates was correlated between orthologous intervals.

**Conclusions:**

We have developed a first-generation bighorn sheep linkage map that will facilitate future studies of the genetic architecture of trait variation in this species. While domestication has been hypothesized to be responsible for the elevated mean recombination rate observed in domestic sheep, our results suggest that it is a characteristic of *Ovis *species. However, domestication may have played a role in altering patterns of heterochiasmy. Finally, we found that interval-specific patterns of sexual dimorphism were preserved among closely related *Ovis *species, possibly due to the conserved position of these intervals relative to the centromeres and telomeres. This study exemplifies how transferring genomic resources from domesticated species to close wild relative can benefit evolutionary ecologists while providing insights into the evolution of genomic structure and recombination rates of domesticated species.

## Background

The construction of genetic linkage maps in model organisms and domesticated species enables studies of the genetic architecture of trait variation and genome evolution. However, such resources for free-living populations of non-model species are still rare because it is difficult to acquire large enough pedigrees and associated sets of molecular markers [1,2]. The utility of genetic linkage maps developed using pedigreed wild populations has been demonstrated by pioneering studies on the genetic architecture of trait variation [3-8], genetic constraints [9] and patterns of linkage disequilibrium [10,11] under semi-natural settings. Yet, we still know very little about these specific topics and the potential to address a variety of additional subjects remains largely unexploited [12]. The development of linkage maps for additional natural populations is therefore clearly desirable.

The bighorn sheep (*Ovis canadensis*), a mountain ungulate inhabiting western North America [13], is one species for which linkage map construction using free-living individuals is possible. DNA samples from intensively studied pedigreed populations have been collected over many decades by field biologists (e.g. [14,15]) and a large set of polymorphic microsatellite markers was recently derived from domestic sheep genomic resources [16]. A bighorn sheep linkage map would enable one to dissect the molecular genetic basis of fitness-related traits, study the molecular basis of inbreeding depression and genetic rescue [14], and potentially reveal the molecular genetic basis of human-influenced evolution [17].

In addition to generating species-specific research opportunities, a bighorn sheep map would shed light on the levels of genomic re-organization between bighorn and domestic sheep. While few differences are expected between these species due to their recent divergence (~3 million years [18]), shared karyotype [19] and ability to produce fertile hybrids [20], enough time has elapsed for rearrangements to accumulate [21]. For example, numerous small-scale rearrangements have been documented between domestic sheep and the slightly more genetically distant domestic goat, *Capra hircus *[22], which can also interbreed with domestic sheep [23]. Reorganization has also been observed among domestic sheep breeds [4,24]. A bighorn sheep linkage map could therefore be used to detect recent chromosomal rearrangements in sheep species and would help with inferring ancestral marker order for regions showing intra-specific variation.

While genome structure is anticipated to be similar between closely related sheep species, expectations for sex-averaged and sex-specific recombination rates are less clear. This is because domestication may have led to an increase in recombination rates and unusual male-biased heterochiasmy in domestic sheep [25,26]. However, the role of domestication in the evolution of mammalian recombination rates remains unclear due to the absence of data on wild relatives [27,28]. A bighorn sheep linkage map would enable such a comparison and help to determine if domestication played a role in the evolution of the atypical recombination patterns seen in domestic sheep.

In this article, we report on the development of a first-generation bighorn sheep genetic linkage map based on the genotyping of 252 polymorphic microsatellites in 498 animals from two pedigreed wild populations: National Bison Range (NBR), Montana, USA [14], and Ram Mountain (RM), Alberta, Canada [15]. The availability of multiple mapping populations permitted a comparison of intra-specific variability in map characteristics as well as the construction of a more contiguous map that should in principle be more representative of the species as a whole. Marker synteny and order were then compared between bighorn sheep and domestic sheep to test for recent chromosomal rearrangements. Finally, we contrasted intervals between species in terms of sex-averaged length and sexual dimorphism to gain insights into the impacts of domestication on the evolution of mammalian recombination rates.

## Results

### Genotyping success and marker polymorphism

Genotyping success was high (~95%) in both populations and is summarised in Table 1 with additional details available in Additional file 1: List of markers, map position and variability. Marker diversity (number of alleles and observed heterozygosity) and the number of informative meioses tended to be greater in the NBR population despite a smaller number of genotyped individuals.

**Table 1:** Marker variability in bighorn sheep mapping populations (range and mean ± 1 SD)

	National Bison Range	Ram Mountain
Marker typing success (%)	42.0 - 100 (95.8 ± 7.8)	52.5 - 100 (94.7 ± 8.8)
Number of alleles	2 - 12 (5.40 ± 1.89)	2 - 12 (4.65 ± 1.73)
Observed heterozygosity	0.06 - 0.90 (0.66 ± 0.13)	0.14 - 0.84 (0.60 ± 0.15)
Total informative meiosis	16 - 310 (225.9 ± 54.4)	42 - 285 (171.3 ± 54.1)
Female informative meiosis	15 - 146 (106.3 ± 25.6)	20 - 142 (83.2 ± 26.6)
Male informative meiosis	1 - 181 (118.1 ± 31.6)	18 - 154 (86.4 ± 28.9)

### Population-specific maps

Linkage analysis for population-specific datasets yielded very similar outcomes. For this reason, only salient features of these maps are presented here while specific details are made available in Additional file 1 and Additional file 2: Comparison of bighorn sheep population-specific maps. In brief, all markers assigned to a linkage group (LG) appeared to be part of the same chromosome in both populations. Map contiguity was slightly greater in the NBR map, with 230 markers distributed along 29 LGs compared to 232 markers distributed along 34 LGs in the RM map. The NBR sex-averaged map spanned 2910 cM while the RM sex-averaged map spanned 2581.4 cM. For both populations, while the overall female autosomal map was longer than the equivalent male map (ratio of 1.13 in NBR and 1.06 in RM), two chromosomes (5 and 15) had longer male maps than female maps. In addition, NBR linkage groups 10, 21, 24 and 25 were longer in the male map while RM linkage groups 2a, 2b, 3c, 8a, 9, 11, 12, 13, 15, 16a, 18, and 19 were longer in the male map.

The most likely marker order differed between maps in only one instance. This involved two tightly linked markers on chromosome 1 (MCM137 and BM7145) for which order was reversed between maps. However, while support for the inferred order was moderate in the NBR map (log_10 _likelihood difference of 2.02, MCM137-BM7145, 0.47 cM), support for the alternate order in the RM map was weak (log_10 _likelihood difference of 0.34, BM7145-MCM137, 0.86 cM).

A comparison of the intervals present in both maps revealed that localized sex-averaged recombination rates were generally similar between populations (r^2 ^= 0.61, Additional file 3: Comparison of intervals present in both population-specific bighorn sheep maps). The sum of these intervals was accordingly similar (2382.9 cM in NBR vs. 2427.8 cM in RM). In both populations, intervals were more often longer in the female map than in the male map (102 vs. 61 in NBR and 92 vs. 72 in RM) but only significantly so in the NBR population (NBR, p < 0.01; RM, p = 0.14). Sexual dimorphism in interval length (sexual dimorphism index, SDI) was significantly more often in the same direction than not (94 out of 160, p < 0.05). However, interval-specific SDI was only weakly correlated between populations (r^2 ^= 0.03, 95% CI = 0 - 0.14).

### Integrated bighorn sheep map

**Figure 1:**
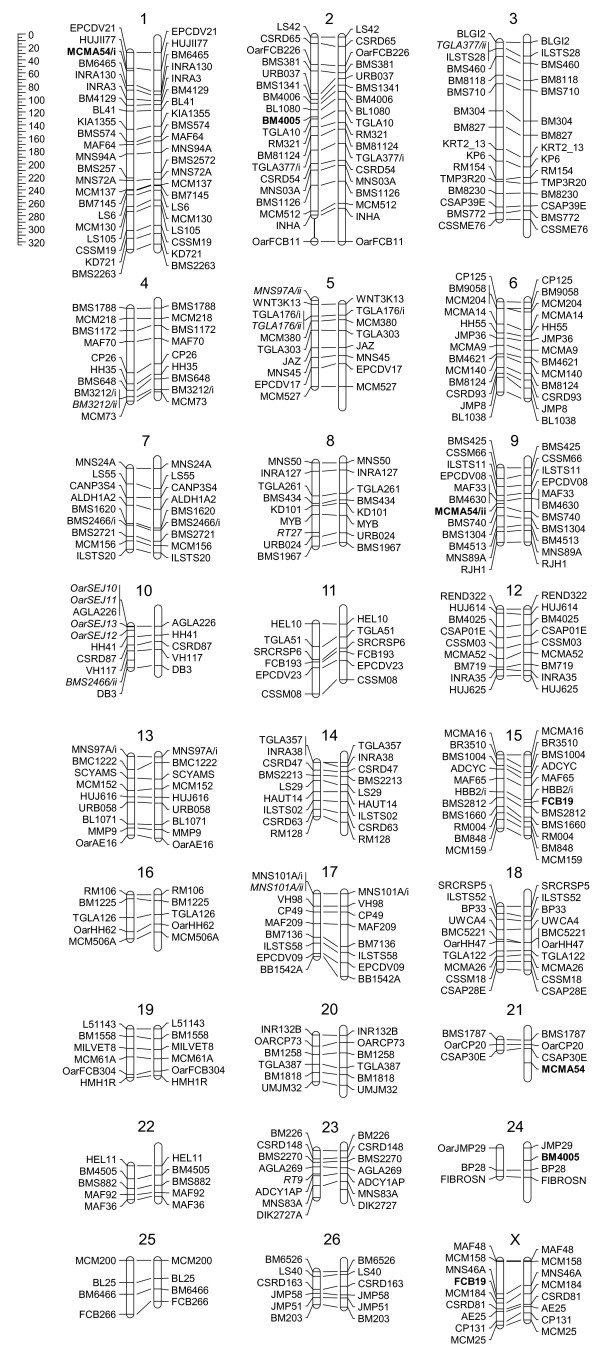
**Bighorn sheep sex-average linkage map compared with the domestic sheep IMF map**. For each chromosome, the bighorn sheep linkage groups (LGs) are on the left while the domestic sheep LGs are on the right. Lines connect orthologous loci. Markers not mapping to the same location in the two species are in bold while markers only mapped in bighorn sheep are italicized. The thin vertical line connecting OarFCB11to chromosome 2 indicates that this marker was assigned to that chromosome but was excluded from the linkage analysis for being more than 50 centimorgans (cM) away from the closest neighbouring marker. That interval was not included in the total map length estimate and its length in the figure is arbitrary. The ruler at the top left corner represents a cM scale.

Combining the two datasets in a single linkage analysis produced a highly contiguous map (Figure 1, Additional file 1). In that analysis, 247 markers were assigned to 27 LGs representing all ovine autosomes and the X chromosome. Since 7 markers were perfectly linked to another marker, the map only truly depicted the locations of 240 unique mapped positions for an average of 8.9 ± 4.3 loci per chromosome. Sex-averaged intervals were on average 14.3 ± 9.1 cM long and usually shorter than 30 cM (Table 2, Additional file 1). The sex-limited and pseudo-autosomal regions of chromosome X were separated by slightly more than 50 cM in the sex-averaged map due to an absence of linkage in the male map but we decided to leave the LG intact due to evidence for tighter linkage (21.5 cM) in the female map. OarFCB11 was excluded from chromosome 2 because it was estimated to be more than 50 cM away from its closest neighbouring marker (INHA). BMS1247, BMS1948 and HBB2/ii could not be assigned to a chromosome while GHRHR was excluded due to having too few informative meioses. The length of the complete sex-averaged map was 3050.9 cM while the autosomal female and male maps were 3166.1 cM and 2832.2 cM long (1.12 ratio), respectively. Intervals were significantly more often longer in the female map than in the male map (119 vs. 87, p < 0.05), however four chromosomes (5, 15, 18 and 24) had longer male than female chromosome maps.

**Table 2:** Descriptive statistics for the integrated bighorn sheep map

			Map length (cM)			No. of intervals (Sex-averaged length)		
**Linkage group**	**No. of markers**	**No. of intervals**	**Sex-averaged**	**Female**	**Male**	**0 - 15 cM**	**15 - 30 cM**	**> 30 cM**

1	22	21	302.8	326.3	284.9	13	7	1
2	18	17	274.3*	290.9*	264*	11	3	3
3	16	15	272.7	303.5	250.1	7	6	2
4	10	8	142.7	167.6	124	3	4	1
5	10	8	132.8	125.2	148.1	3	5	0
6	13	11	138.9	148.3	135.4	8	3	0
7	9	8	125.3	136.8	116.8	4	4	0
8	9	8	127.9	155.6	122.9	6	1	1
9	12	10	115.5	122.5	109.9	7	3	0
10	10	8	64.2	65	64	8	0	0
11	6	5	108.2	118.3	99.2	2	1	2
12	9	8	102.9	107.3	99.5	4	4	0
13	9	8	120.6	122.5	119.7	5	2	1
14	9	7	82.5	92.2	75.3	6	1	0
15	11	10	112.8	110.5	118.8	9	1	0
16	5	4	67.6	77.4	62.3	2	2	0
17	9	7	97.3	100.5	97.3	3	4	0
18	10	9	96.9	94.4	97.4	6	3	0
19	6	5	75.5	75.5	74.8	3	2	0
20	6	5	71.5	77.9	66.3	4	1	0
21	3	2	16.3	16.6	16	2	0	0
22	5	4	51.9	60.9	45.8	3	1	0
23	8	7	71.9	82	63.9	6	1	0
24	3	2	44	41.3	47.9	1	0	1
25	4	3	83.3	89	80.8	0	1	2
26	6	5	51.3	58	46	4	1	0
X	9	8	99.2	170.6	1.3**	7	0	1
Total	247	213	3050.8	3336.6	2832.4	137	61	15

*excluding FCB11 which is more than 50 cM away

**Pseudo-autosomal region

### Comparison of bighorn sheep and domestic sheep maps

Synteny was highly similar between the bighorn sheep and the domestic sheep International Mapping Flock (IMF) maps with only three observed differences (Figure 1). First, FCB19 mapped to chromosome X in bighorn sheep but to chromosome 15 in domestic sheep. Second, BM4005 mapped to chromosome 2 in bighorn sheep but to chromosome 24 in domestic sheep. Finally, neither of the two markers amplified in bighorn sheep with the primer pair used for MCMA54 in domestic sheep mapped to the location of this marker predicted from the IMF map (chromosome 21). Instead, MCMA54/i and MCMA54/ii mapped to bighorn sheep chromosomes 1 and 9, respectively. For the three other primer pairs which amplified two unlinked markers in bighorn sheep (TGLA377, BMS2466, MNS97A), one of the markers mapped to its predicted position while the other mapped to a different chromosome (TGLA377/ii, MNS97A/ii and BMS2466/ii were assigned to chromosome 3, 5 and 10, respectively). One additional putative difference between species was observed on chromosome 10 for markers not mapped in the IMF but mapped in Soay sheep, a feral domestic sheep breed [8]. The most likely order for this region in bighorn sheep was OarSEJ10, OarSEJ11, AGLA226 and OarSEJ13 versus AGLA226, OarSEJ10, OarSEJ11 and OarSEJ13 in Soay sheep. The difference in log_10 _likelihood between marker orders in bighorn sheep was 3.01.

**Figure 2:**
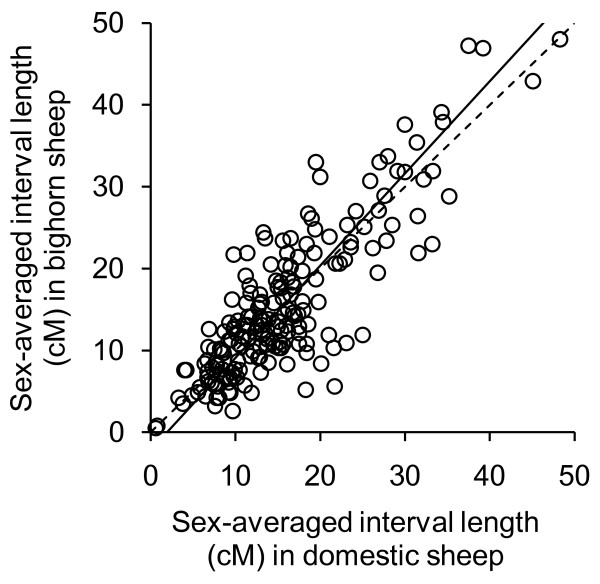
**Comparison of sex-averaged interval length (cM) in bighorn sheep and domestic sheep for 203 pairs of adjacent markers**. The solid line depicts the relationship between bighorn sheep and domestic orthologous intervals (reduced major axis regression, y = 1.14 × - 1.83, r^2 ^= 0.72, 95% CI = 0.60, 0.80) while the dashed line separates intervals larger in bighorn sheep (above line, n = 105) from intervals larger in domestic sheep (below line, n = 98).

The length of orthologous intervals was highly correlated between species (r^2 ^= 0.71, p < 0.01, Figure 2, Additional file 4: Comparison of intervals present in bighorn sheep and domestic sheep maps) and their sum very similar (3044 cM in bighorn sheep vs. 3001 cM in domestic sheep; a difference of ~1.5%). This excluded the intervals located at the tip of bighorn sheep chromosomes 5 (MNS97A/ii to WNT3K13, 6 cM) and 10 (OarSEJ10 to AGLA226, 0.5 cM) that have no equivalent in the IMF map. Intervals did not tend to be larger in one species than the other (105 larger in domestic sheep vs. 98 larger in bighorn sheep, p = 0.67). Based on coverage of these intervals in the version 4.7 IMF map, we estimated the current genome coverage by the integrated map in bighorn sheep to correspond to ~ 84% of the domestic sheep linkage map.

**Figure 3:**
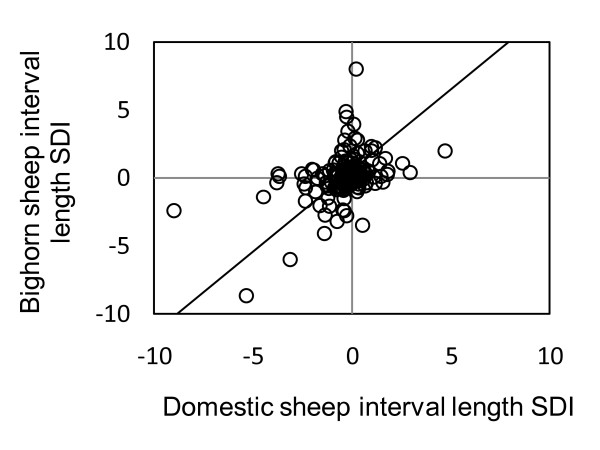
**Comparison of sexual dimorphism (SDI) in interval length between orthologous bighorn sheep and domestic sheep intervals**. 192 intervals between adjacent markers were compared (reduced major axis regression, y = 0.95 × + 0.42, r^2 ^= 0.21, 95% CI = 0.09, 0.37). SDI values are positive when intervals are larger in females and negative when intervals are larger in males.

Mean SDI in length (± 1 SD) considering only orthologous intervals was 0.10 ± 1.27 in bighorn sheep and -0.34 ± 1.33 in domestic sheep. The positive mean SDI in bighorn sheep reflected a tendency for larger intervals in the female map (114 out of 197, p < 0.05) while the negative mean SDI in domestic sheep indicated a tendency for larger intervals in the male map (120 out of 197, p < 0.01). Interval-specific SDI was significantly correlated between species (r^2 ^= 0.21, p < 0.01, Figure 3). The intercept and slope were both significantly positive (intercept ± 1 SE: 0.42 ± 0.08, p < 0.01; slope: 0.95 ± 0.06, p < 0.01). In general, SDI values in bighorn sheep tended to be greater than in domestic sheep (124 times out of 189, P < 0.001).

## Discussion

As expected, marker synteny and order were generally congruent between bighorn sheep maps. This suggests that our dataset was mostly free of errors and justified combining individual maps. The NBR population was generally more informative than the RM population. This was likely a consequence of the more complete NBR pedigree combined with greater marker variability resulting from recent admixture [14,16]. Nonetheless, information provided by both populations was generally complementary and ultimately allowed construction of a highly contiguous map covering approximately 84% of the species genome. This is greater coverage than for a similar map for free-ranging red deer (*Cervus elephus*, 39% [3]) and almost on a par with one for Soay sheep (*Ovis aries*, 90% [4]) for which virtually all genetic resources developed for domestic sheep can be used. The coverage of our map is therefore similar to a first-generation map for a domestic species and outstanding for a wild species.

Recombination fractions were very similar between bighorn sheep populations. Combining pedigrees into a single analysis therefore likely resulted in map distances that were generally representative of the species as a whole. While genuine intra-specific differences may exist in map distances, the integrated map is likely to more accurately depict recombination fractions of individual populations than the estimates derived from the population-specific maps. This is because interval estimates for population-specific maps were often based on relatively few informative meioses, especially in the RM population. Relying on distances from the integrated map in future downstream population-specific studies is therefore advisable.

As predicted, marker synteny and order were generally congruent between the *Ovis *species maps. This is in line with the expectation of 1 to 2 rearrangements per million years in most mammalian lineages [20]. However, it has to be acknowledged that marker coverage was generally too sparse to detect subtle rearrangements. Cross-species comparison was also made difficult by the fact that some primer pairs amplified two loci. For example, BM4005 mapped to different locations in each species but we are aware of a second locus for BM4005 in bighorn sheep that could not be reliably genotyped. Since primers for BM4005 are also known to amplify multiple sets of bands in domestic sheep [29], the BM4005 loci mapped in the two species are probably not orthologous. Similarly, FCB19 mapped to chromosome X in bighorn sheep but to chromosome 15 in domestic sheep. This marker is definitely autosomal in domestic sheep given that a fraction of males are undoubtedly heterozygous (J. Maddox, unpublished data) so the discrepancy in map location is not spurious. However, FCB19 markers amplified in the two species might not be orthologous given that a single primer pair can amplify multiple markers. In contrast, convincing evidence for cross-species rearrangement came from the primers used to amplify MCMA54 in domestic sheep. In that case, neither of the two markers amplified using this primer pair in bighorn sheep mapped to the location of the MCMA54 locus in domestic sheep (the MCMA54 primers amplified two band sets that both mapped to chromosome 21 in domestic sheep vs. 1 and 9 in bighorn sheep). The comparison of our map with the Soay sheep map [8] also suggested the presence of a minor rearrangement on chromosome 10. While some of these cases may depict genuine rearrangements, it is clear from this study that the organization of the two species genomes is very similar.

Genomic analysis in a close relative of domestic sheep offered the opportunity to infer ancestral marker order for chromosomal regions showing variation among domestic sheep breeds [4,26]. For chromosome 1, the order of two loci located in the rearranged region (MCM137 and BM7145) was similar between the NBR and the IMF maps [24]. On the other hand, the most likely marker order in the RM map was similar to an alternate order documented in Soay sheep [24]. Inferred marker orders were arguably not significantly more likely than the alternate orders. Yet, it is worth noting that this chromosomal region was the only one for which the most likely marker order differed between bighorn sheep maps. If both orders are present in bighorn sheep, it would mean that this region is either prone to rearrangements or that polymorphism in marker order originated millions of years ago rather than recently as hypothesised by McRae and Beraldi [24]. For a second putatively varying region located on chromosome 12 [4], we only successfully amplified one (BM4025) of the two markers used to infer rearrangement in domestic sheep (BM4025 and TGLA53). However, marker order in bighorn sheep for that region appeared to be the same as in the IMF map based on a marker located only 2 cM away from TGLA53 in domestic sheep (CSAP01E). Therefore, the IMF appeared to portray the ancestral marker order.

The comparison of orthologous intervals suggested high similarity in localized sex-averaged recombination rates between the *Ovis *species. While the near perfect concordance in total map length (~1%) may be coincidental, given that variation in the order of 10% has been documented among domestic sheep breeds [4], it nonetheless strongly suggests little difference between species. Assuming that results were not unduly biased by missing and erroneous genotypes (which can be a concern when using CRI-MAP in complex pedigrees [2,30]), it appears that the elevated recombination rates observed in domestic sheep are a characteristic of *Ovis *species rather than a consequence of domestication. Alternatively, recombination rates may have increased rapidly in both species since their recent divergence as a consequence of domestication in domestic sheep and for a different reason in bighorn sheep. But, this later explanation seems unlikely since the evolution of mean recombination rates in mammals is generally slow and most likely governed by neutral processes [28].

Contrary to what has been found for domestic sheep, recombination rates in bighorn sheep tended to be greater in females than in males. The unusual pattern observed in domestic sheep therefore appeared to be species-specific. This finding is not overly surprising given the low phylogenetic inertia of the trait [31]. The magnitude of heterochiasmy in sheep species is also arguably modest when compared with species such as the saltwater crocodile (*Crocodylus porosus*, ratio of 5.7:1 [32]) or the zebrafish (*Danio rerio*, ratio of 2.74:1 [33]). Yet, the presence of male-biased recombination in domestic sheep remains puzzling given that recombination in placental mammals is generally female-biased [26]. An intuitive explanation is that altered sex-specific recombination patterns in domesticated mammals (cattle are also atypical, exhibiting no heterochiasmy [34]) might be an incidental result of strong artificial selection during the process of domestication. Alternatively, the unusual heterochiasmy pattern documented in the domestic IMF might simply be an artefact resulting from the facts that the population size was small, all sires descended from a single grand-sire and there were only three maternal grandsires compared to 13 granddams. Knowing that recombination rates can vary substantially among individuals, and that such differences can have a large genetic component (e.g. [35]), it could be that the paternal grand-sire was characterised by an uncommonly high recombination rate breeding value and/or that some of the maternal grandsires were characterised by uncommonly small recombination rate breeding values (assuming that male and female recombination rates are positively genetically correlated [36]). A comparison of sex-specific recombination rates in additional domestic sheep pedigrees might answer this question.

**Figure 4:**
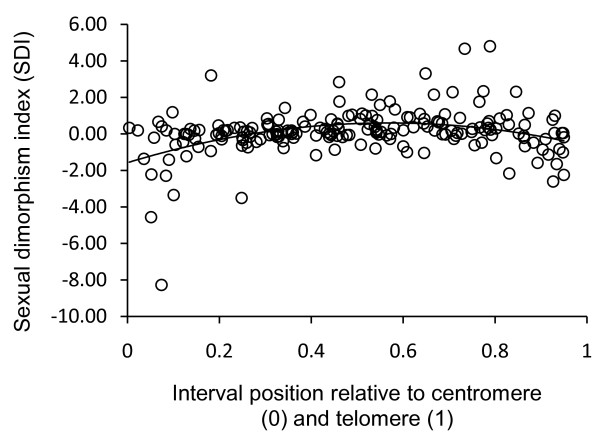
**Relationship between interval length sexual dimorphism (SDI) and relative distance from centromeres and telomeres in bighorn sheep**. The location of each interval relative to centromeres (0) and telomeres (1) were inferred using the position of orthologous intervals in the domestic sheep IMF map version 4.7. The fitted curve is a second order polynomial (r^2 ^= 0.16, quadratic term fitted in a linear model, p < 0.001).

As in other taxa (e.g. [37,38]), great variability was observed in patterns of heterochiasmy across and along chromosomes. For example, recombination appeared to be male-biased for a few chromosomes despite the presence of a genome-wide tendency for greater recombination in females. However, no clear pattern emerged at the chromosomal level with the NBR and RM maps yielding mainly inconsistent results. At the interval scale, patterns of sexual dimorphism were conserved across populations and species. This means, for example, that genomic regions characterized by low SDI values in one species were mirrored by similarly low SDI values in the other species. This could be due to conserved sex-specific and/or sex-biased recombination hot-spots. However, fine-scale analyses of recombination rates in other pairs of closely related species (e.g. human and chimpanzee [39,40]) suggest that this is unlikely at the inter-specific level. Inter-specific congruence in localized recombination rate sexual dimorphism could also be due to the position of intervals along chromosomes relative to centromeres and telomeres, irrespective of the exact location of individual hot-spots. For example, in humans, recombination tends to be greater in females near centromeres but greater in males near telomeres (reviewed in [41]). In domestic sheep, recombination in telomeric and centromeric regions is usually greater in males (J Maddox, unpublished data). To verify if a similar pattern was also present in bighorn sheep, we contrasted bighorn sheep interval-specific SDI to the relative distance of these intervals from centromeres and telomeres inferred from the location of these intervals in the IMF map. A pattern similar to that seen in domestic sheep was observed, with recombination being greater in males near centromeres and telomeres while being greater in females in more central parts of chromosomes (Figure 4).

## Conclusion

We constructed a first-generation bighorn sheep linkage map using DNA from two wild pedigreed population and genomic resources originally developed for domestic sheep. Since bighorn sheep and domestic sheep genomes are very similar, future efforts to increase marker density in specific chromosomal regions should be relatively straightforward. This could be achieved using bighorn sheep single nucleotide polymorphism (SNP) markers recently discovered using the OvineSNP50 Beadchip [42], additional microsatellites already mapped in domestic sheep and/or by taking advantage of the recently acquired domestic sheep genome sequence [43] to develop novel markers. The high similarity between the genomes of the two species should also greatly facilitate future efforts to assemble a bighorn sheep genome sequence and to develop additional SNPs.

The main reason for developing genomic resources in bighorn sheep is to allow studies of complex trait genetic architecture and evolution under natural settings. In the NBR population, genomic resources will enable investigations into the genetic basis of fitness, inbreeding depression and genetic rescue [14]. In RM, it will be possible to study the genetic architecture of additional traits including body mass, horn size and animal personality [15,44]. Finally, genomic information could eventually be combined with population genetic approaches to study adaptive population differentiation [45], especially in the context of parasitism [46] and selective harvesting [47].

While resources developed for domestic sheep are obviously highly useful to bighorn sheep research, genomic research in bighorn sheep can also yield valuable information through comparisons with domestic sheep in return. For example, we have demonstrated how linkage mapping in bighorn sheep can be used to infer ancestral marker order in domestic sheep. Also, by comparing the domestic sheep map with the map of a close wild relative, we were able to determine that the elevated recombination rates observed in domestic sheep were likely a characteristic of *Ovis *species while the unusual male-biased heterochiasmy might have been a consequence of domestication. Finally, we have demonstrated that interval-specific patterns of sexual dimorphism could be conserved among closely related species, possibly due to the position of these intervals relative to centromeres and telomeres.

## Methods

### Study populations

#### National Bison Range

The NBR population was established by transplanting four rams and eight potentially pregnant ewes from Banff National Park (Alberta, Canada) in 1922 [14]. The population remained isolated until the introduction of five rams in 1985 and 10 sheep (three rams and seven ewes) from 1990 to 1994. Fourteen of these more recently introduced animals were derived from a native Montana population (Sun River) while one ewe was from a native Wyoming herd (Whisky Basin). Individuals from these latter introductions were highly successful [14], resulting in relatively high levels genetic diversity and linkage disequilibrium [16]. All sheep were individually recognizable through physical characteristics from 1979 onward and collection of blood/tissue samples for genetic analysis began in 1988. Our analyses included a combination of descendants from the original introduction, recent immigrants and admixed individuals.

#### Ram Mountain

The RM population is native to a small isolated mountain range located about 50 km east of the Canadian Rockies in Alberta, Canada [15]. Immigration and emigration is highly restricted and mainly limited to exchanges with a smaller unmonitored herd located on the same mountain range. Animals were captured in a corral trap baited with salt and marked with unique tags as lambs or yearlings. Population monitoring began in the early 1970s and collection of hair/blood/skin samples for genetic analysis began in 1988.

### Mapping pedigrees

In both populations, parentage was originally determined with ~30 microsatellite loci using the 95% confidence threshold in Cervus [48]. For the RM population, the markers used are presented in [15] and references therein. For the NBR population, the markers included the ones listed in [14] as well as BL25, BM1225, BM1818, BM4505, BM4630, BM848, BMC1222, MAF92, OarJMP29, TGLA126, TGLA387, EPCDV21, MCMA54/i and MCMA54/ii. Laboratory methods are detailed in [14,15] and references therein. References for primer sequences are available in Additional file 1. Reconstructed pedigrees were used to identify animals expected to contribute the most information for linkage mapping purposes (e.g. large sibships and their parents) and these animals were then genotyped at more than 200 microsatellite loci (details below). Once genome-wide genotypes were obtained, animals for which genotyping success was low (< 65%) were discarded and the pedigrees were updated based on new parentage analyses. Following these steps, no more than 2-3 mismatches were observed between parent-offspring pairs. The software Pedcheck [49] was then used to identify Mendelian inconsistencies which were corrected when possible or otherwise eliminated by deleting the genotypes of the individuals involved. The resulting NBR and RM mapping pedigrees spanned seven and six generations and included 212 and 286 related individuals, respectively. Pedigree illustrations produced using Pedigree Viewer [50] are available in Additional file 5: Bighorn sheep mapping pedigrees. The NBR pedigree contained 184 paternal links (42 sires, mean ± 1 SD of 4.4 ± 3.5 offspring per sire) and 173 maternal links (51 dams, 3.4 ± 2.1 offspring per dam). The RM pedigree consisted of 168 paternal links (43 sires, 3.9 ± 3.3 offspring per sire) and 172 maternal links (71 dams, 2.4 ± 1.3 offspring per dam).

### Microsatellite selection and genotyping

In addition to markers used for the initial pedigree reconstruction, microsatellites putatively distributed throughout the genome of our focal species were identified using the domestic sheep IMF map version 4.7 [16,51]. Markers were selected based on their predicted genomic location and level of polymorphism (assessed in ~30 individuals/population) with the aim of optimising genomic coverage and meiotic information. Most but not all markers were typed in both populations. Eleven markers were only genotyped in the NBR population while 17 were only typed in the RM population (see Additional file 1). Laboratory methods are available in [16] and references for primer sequences [8,16,51-55] are presented in Additional file 1. In total, 252 markers, amplified using 244 pairs of primers (8 primer pairs amplified two markers: BM3212, BMS2466, HBB2, MCMA54, MNS97A, MNS101A, TGLA176, TGLA377), were included in the linkage analysis. Descriptive statistics (typing success, number of alleles and observed heterozygosity) were obtained using MSA 4.05 [56].

### Linkage analysis

We constructed population-specific linkage maps as well as an integrated map where populations were treated as independent families using CRI-MAP [57]. The same construction procedure was used for all maps. First, two-point linkage analyses were performed for all pairs of markers assuming equal recombination rates between the sexes using a modified version of CRI-MAP developed by Liu and Grosz [58]. The program AUTOGROUP [58] was then used to identify sets of markers likely residing in the same LG (pairwise LOD scores > 4). For markers unassigned to a LG following that analysis, two-point LOD scores were inspected and in cases where the most likely linkage was with a marker known to be adjacent in the domestic sheep IMF map, the marker was assumed to be part of the same LG in bighorn sheep. In cases where multiple bighorn sheep LGs were composed of markers known to be part of the same chromosome in domestic sheep, two-point LOD scores between markers residing at the end of each bighorn sheep LG were inspected and linkage was assumed when the LOD scores were among the highest for these respective markers. For each putative LG, the most likely marker order was recovered using the BUILD and FLIPSn options of a CRI-MAP version recently developed by Jill Maddox and Ian Evans (2.503) that more efficiently deals with large datasets. Specifically, we first constructed LGs using BUILD and a LOD > 3 threshold. Markers were then successively added to these LGs using less stringent LOD thresholds of 2, 1, 0.5 and 0. The FLIPSn option was then used to compare the likelihood of alternate orders produced by shuffling up to five adjacent loci and markers were re-ordered when a more likely order was identified. Doubtful tight double recombinants were identified using the CHROMPIC option of CRI-MAP and responsible erroneous genotypes were corrected when present. Finally, sex-averaged and sex-specific recombination fractions for individual LGs were estimated using the FIXED option of CRI-MAP 2.503 and transformed to centimorgans (cM) using the Kosambi map function [59]. In cases where estimated sex-averaged intervals were greater than 50 cM, LGs were broken in two and separate analyses were performed for markers on each side of the interval.

### Comparison of linkage maps

In order to assess intra- and inter-specific variability in genomic structure and recombination rates, we compared the NBR and RM linkage maps as well as the bighorn sheep integrated map and domestic sheep IMF map version 4.7. Differences in marker synteny and order were identified by visual inspection. In cases where the most likely marker order differed between populations/species, support for alternate orders was determined by comparing log_10 _likelihoods. The relative sex-averaged length of different maps was compared by summing the length of intervals that were present in both maps (raw data available in Additional file 3 and Additional file 4).

To test for a genome-wide difference in sex-averaged recombination rate between population/species, we used two-tailed sign tests contrasting the number of shared intervals greater in length in one map than in the other. We also used reduced major axis regression to describe the relationship between interval-specific sex-averaged recombination rates between populations/species. The slopes, intercepts and their errors were obtained using the formula from Sokal and Rohlf [60] implemented in the software RMA version 1.17 [61]. Confidence intervals including the ones for correlation coefficients were obtained by performing 10000 bootstraps.

To assess variation in heterochiasmy across populations and species genomes, we quantified sexual dimorphism for individual intervals using the sexual dimorphism index (SDI) of Lovich and Gibbons [62]. This index is considered the best estimator of sexual dimorphism because it is intuitive, linear, symmetrical, and directional [63]. The SDI was obtained by subtracting 1 from the ratio of the largest sex-specific value to the smallest sex-specific value. Following convention, estimates were then made positive when the female value was largest and negative when the male value was largest. We tested for the presence of genome-wide bias in sexual dimorphism using sign tests and described the relationship in interval-specific sexual dimorphism between maps using reduced major axis regression.

Since the length of an interval partly depends on the subset of markers included in a linkage analysis, we assessed the validity of comparing intervals between maps constructed using different number of markers (the domestic sheep IMF map 4.7 contains about 1400 markers). To accomplish this, we repeated cross-species analyses using information derived from additional linkage maps based solely on markers mapped in both species. Results and conclusions were essentially the same as for previous analyses and are therefore not presented. These maps are available in Additional file 6: Bighorn sheep and domestic sheep linkage maps based on shared markers only.

### Ethics

All research protocols were approved by the University of Alberta Animal Use and Care Committee, affiliated with the Canadian Council for Animal Care (Certificate 610901).

## Authors' contributions

JP designed the study, conducted laboratory work, performed data analysis and wrote the manuscript. JTH designed and supervised fieldwork at NBR and performed paternity analyses. CSD implemented laboratory methods and performed laboratory work. JMM participated in laboratory work and data analysis. JFM provided marker information and constructed a domestic sheep linkage map composed solely of markers mapped in bighorn sheep. DWC planned and supervised the study. All co-authors read and commented on draft versions of the manuscript. All authors read and approved the final manuscript.

## Supplementary Material

Additional file 1 **List of markers, map position and variability**. Excel document displaying List of markers, map position and variability. Click here for file

Additional file 2 **Comparison of bighorn sheep population-specific maps**. PDF displaying comparison of bighorn sheep population-specific maps. Click here for file

Additional file 3 **Comparison of intervals present in both population-specific bighorn sheep maps**. XLS file displaying comparison of intervals present in both population-specific bighorn sheep maps. Click here for file

Additional file 4 **Comparison of intervals present in bighorn sheep and domestic sheep maps**. XLS file displaying comparison of intervals present in bighorn sheep and domestic sheep maps. Click here for file

Additional file 5 **Bighorn sheep mapping pedigrees**. PDF file displaying bighorn sheep mapping pedigrees Click here for file

Additional file 6 **Bighorn sheep and domestic sheep linkage maps based on shared markers only**. XLS bighorn sheep and domestic sheep linkage maps based on shared markers only Click here for file
