# Supplementary material for: Genetic linkage map of a wild genome: genomic structure, recombination and sexual dimorphism in bighorn sheep
Source: BMC Genomics. 2010 Sep 28;11:524. doi: 10.1186/1471-2164-11-524 (PMC3091677; doi:10.1186/1471-2164-11-524)
Supplement: Additional file 2 — Comparison of bighorn sheep population-specific maps. PDF displaying comparison of bighorn sheep population-specific maps. [file 1471-2164-11-524-S2.PDF]

Additional file 2: Comparison of bighorn sheep population-specific maps

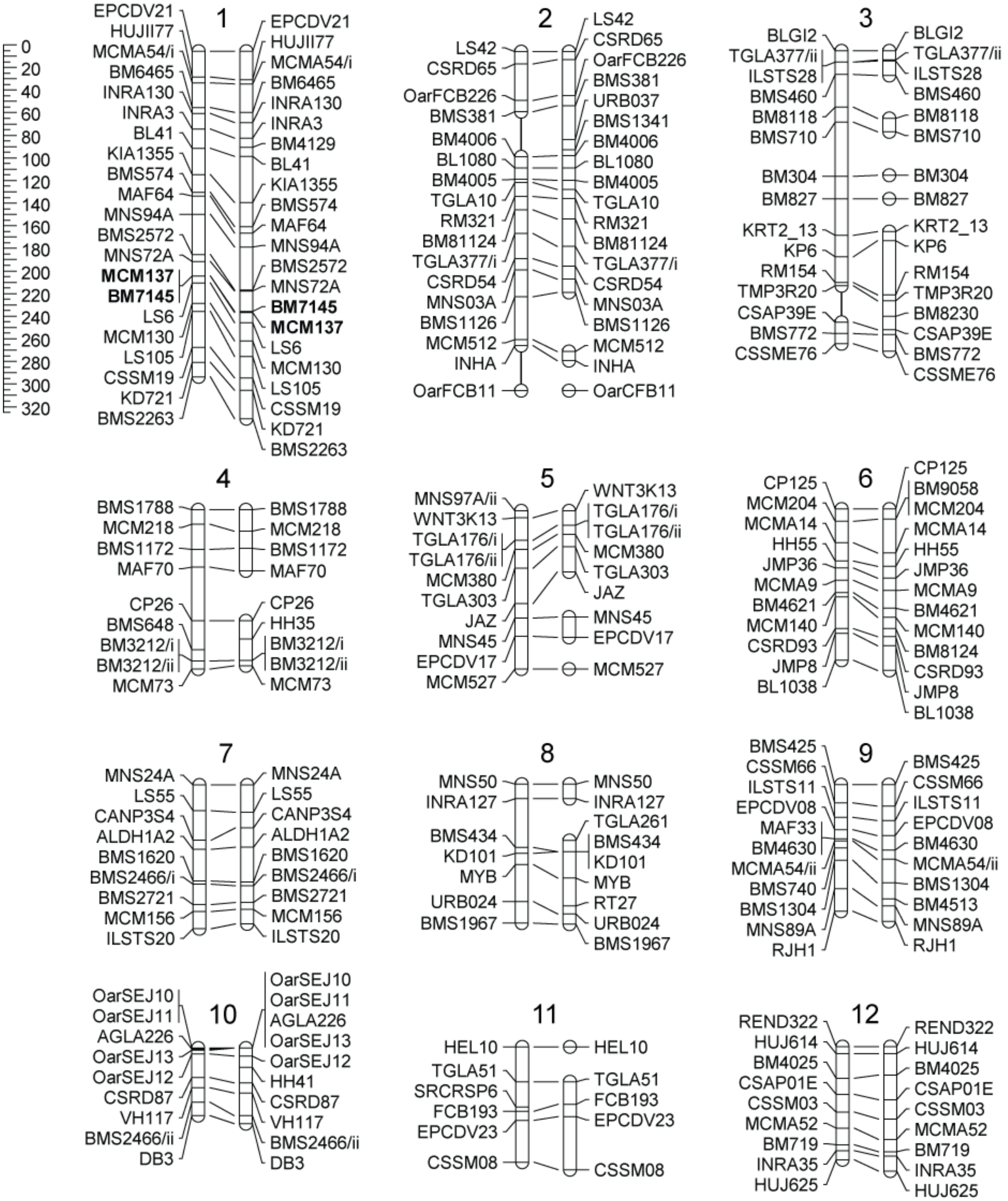

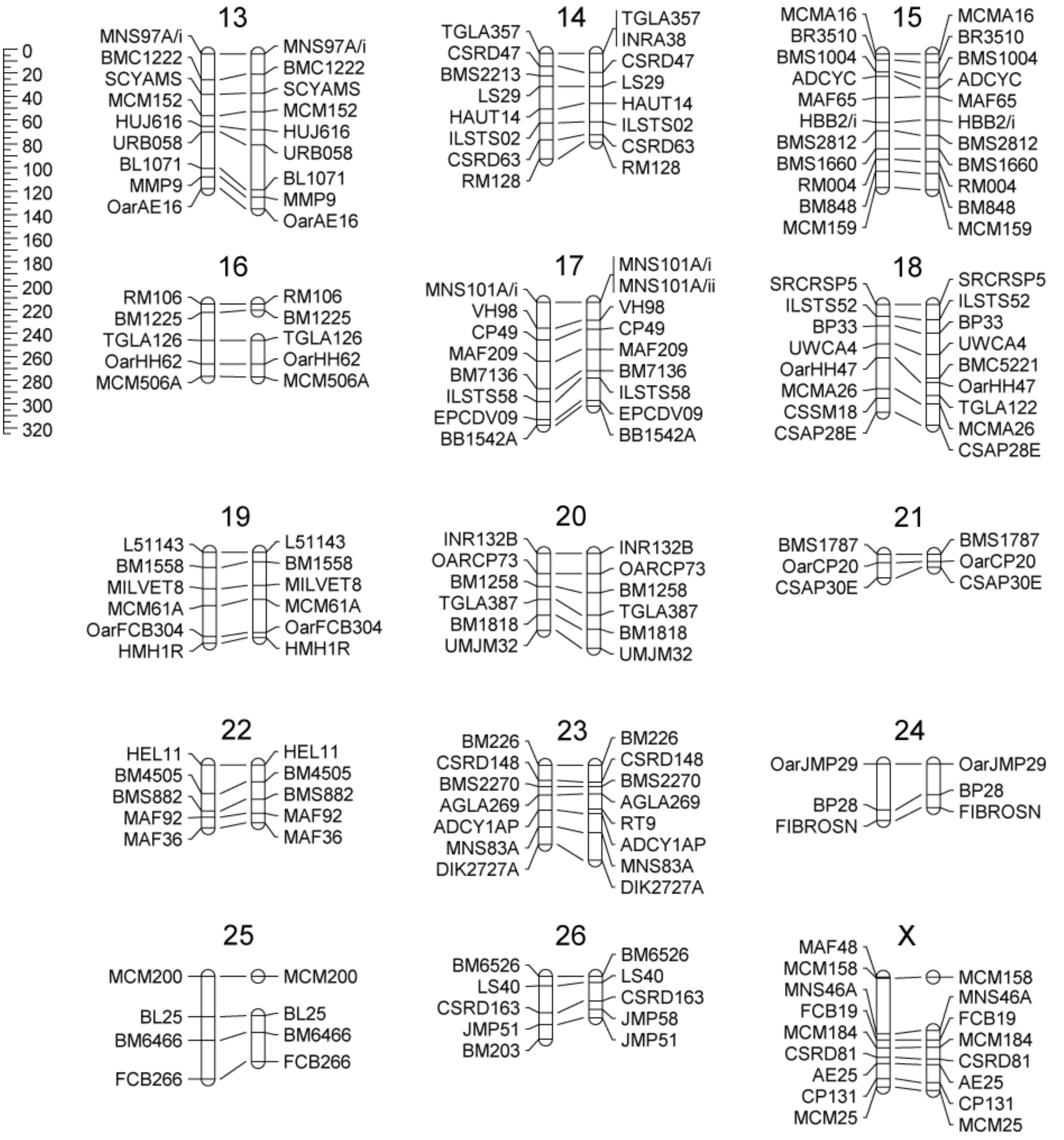


**Comparison of population-specific bighorn sheep linkage maps**. For each chromosome, the National Bison Range linkage groups are on the left while the Ram Mountain linkage groups are on the right. Lines connect homologous loci. Markers not mapping to the same location in the two populations are in bold. Thin vertical lines connecting OarFCB11 to chromosome 2 as well as some linkage groups indicate that linkage was inferred based on LOD scores but that separate

analyses were ultimately performed for both side of the intervals because these intervals were estimated to exceed 50 cM in length. These intervals were not included in the estimation of total map lengths and their length in the figure is arbitrary. The ruler at the top left corner represents a cM scale.
