# Supplementary material for: Genetic linkage map of a wild genome: genomic structure, recombination and sexual dimorphism in bighorn sheep
Source: BMC Genomics. 2010 Sep 28;11:524. doi: 10.1186/1471-2164-11-524 (PMC3091677; doi:10.1186/1471-2164-11-524)
Supplement: Additional file 5 — Bighorn sheep mapping pedigrees. PDF file displaying bighorn sheep mapping pedigrees [file 1471-2164-11-524-S5.PDF]

Additional file 5: Bighorn sheep mapping pedigrees

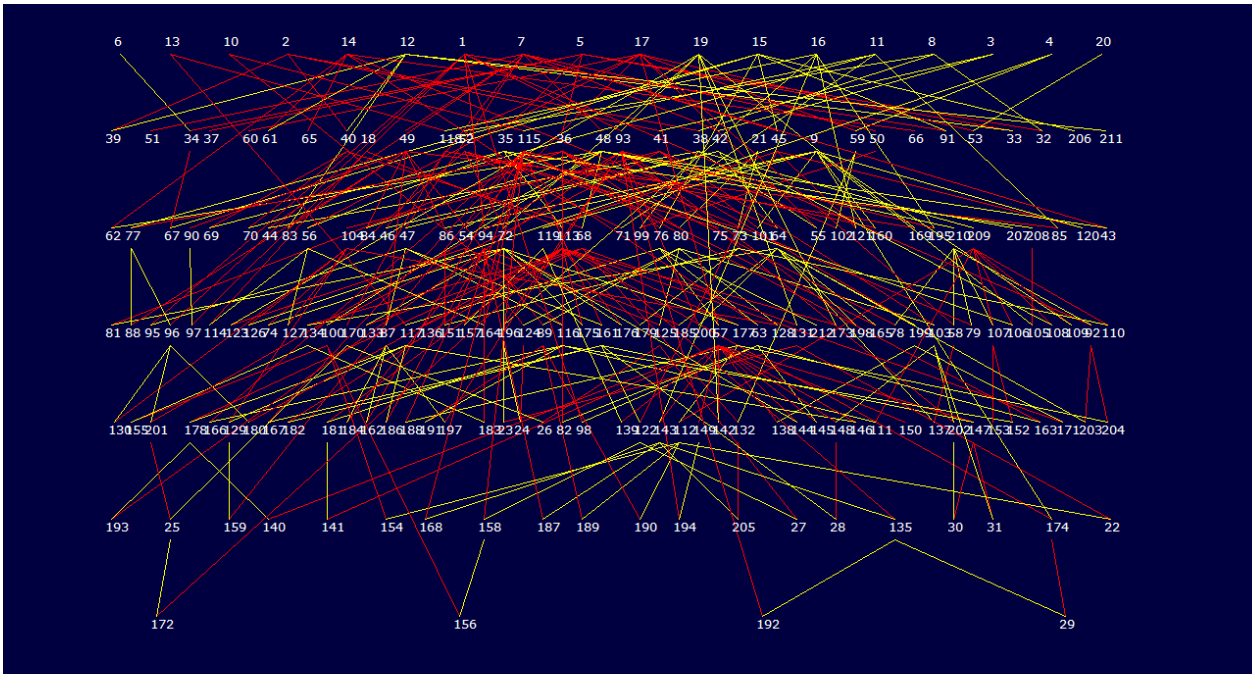

National Bison Range mapping pedigree

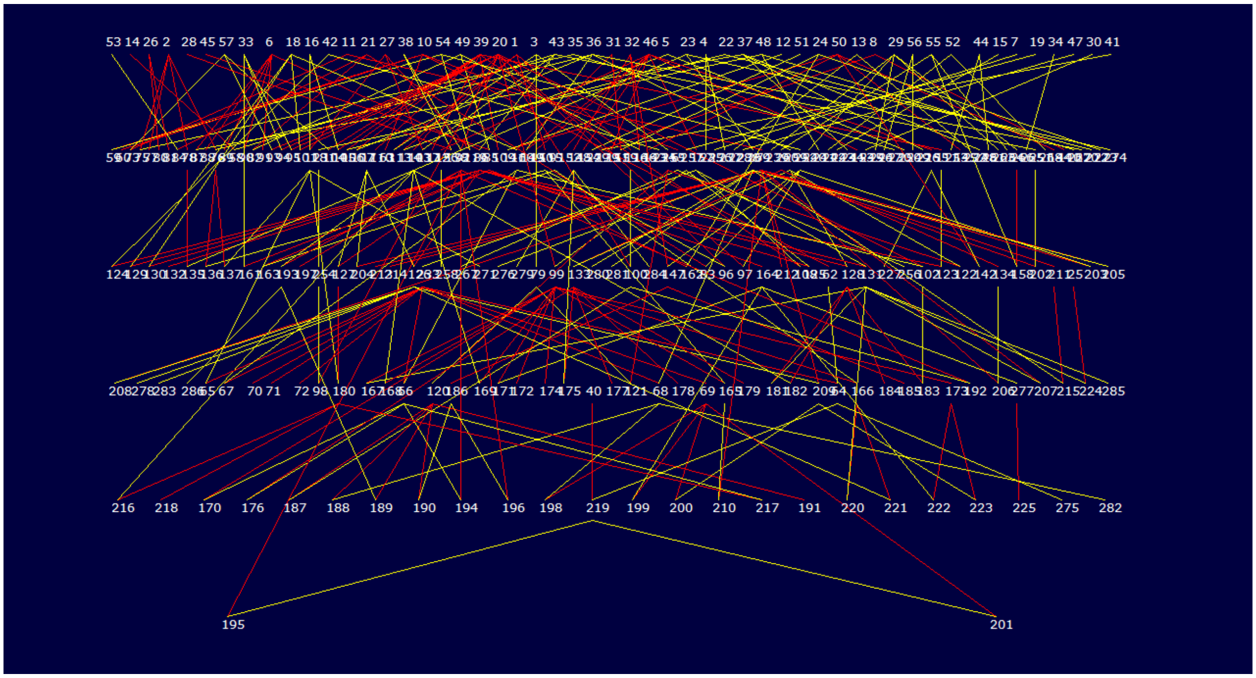

Ram Mountain mapping pedigree
